# Bacteria-mediated tumor immunotherapy *via* photothermally-programmed PD1 expression†

**DOI:** 10.1039/d1na00857a

**Published:** 2022-02-07

**Authors:** Wenxuan Xu, Debao Ren, Zimeng Yu, Jia Hou, Fan Huang, Tingfang Gan, Ping Ji, Cheng Zhang, Lixin Ma, Yunhong Hu

**Affiliations:** State Key Laboratory of Biocatalysis and Enzyme Engineering, Hubei Key Laboratory of Industrial Biotechnology, School of Life Sciences, Hubei University Wuhan 430062 P. R. China malixing@hubu.edu.cn huyunhong@hubu.edu.cn; Department of Chemistry, Wuhan University Wuhan 430072 P. R. China

## Abstract

The special microenvironment of a solid tumor promotes the orientation and colonization of facultative anaerobes. Intratumoral bacterial infection disrupts the local vascular system to form a thrombus, resulting in darkened tumor sites and enhanced near-infrared absorption. Based on this, we constructed thermally-induced bacteria (TIB) to express programmed cell death protein 1 (PD1) at tumor tissue sites. Under laser irradiation, the elevated temperature at the tumor site not only caused damage to tumor cells but also induced the expression of PD1. Expressed PD1 bound to the ligand of PD1 (PD-L1) on the tumor cell surface and facilitated its internalization and reduction, thereby relieving immune suppression in the tumor microenvironment. Through the combined effects of photothermal therapy and immune activation, the ingenious TIB@PD1 approach greatly inhibited the proliferation and metastasis of tumor cells. Therefore, bacteria-based photothermal immunotherapy represents an appealing method for tumor therapy with good specificity and selectivity.

## Abstract 2

A thermally-induced bacteria platform was constructed for oncotherapy. *E. coli* colonized the tumor tissue and form a thrombus. After NIR irradiation, local hyperthermia and heat-induced PD1 inhibit the proliferation and metastasis of tumor cells.

## Graphical abstract

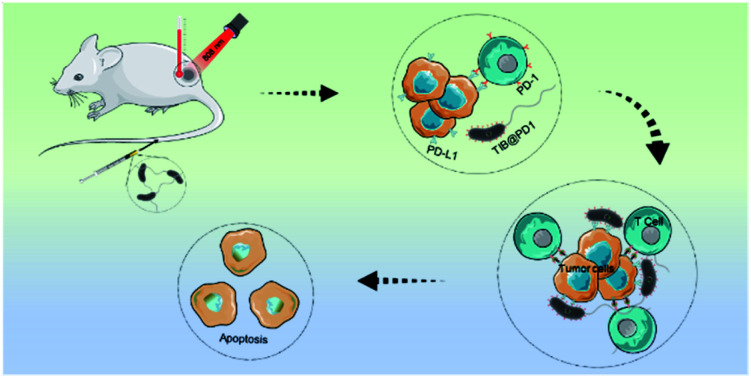

## Introduction

Due to the threat of a malignant tumor, thousands of people's lives and health are affected every year.^1^ At present, surgical resection, radiotherapy, and chemotherapy are known to be the most frequently used methods for tumor treatment, but they are accompanied by a series of problems, such as high risk, serious surgical trauma, obvious side effects, severe pain, and easy relapse.^2,3^ With deepening research into the mechanism of tumor genesis and development, a growing number of targeted tumor drugs and methods, such as immunotherapy, gene therapy, photodynamic therapy, photothermal therapy (PTT), and sonodynamic therapy, have been developed and clinically applied for tumor treatment.^4–9^

Representatively, PTT as a new noninvasive strategy plays an attractive role in tumor treatment. The heat produced by PTT could lead to tumor cell pyroptosis and activate the body's immune system at the same time.^10–12^ Taking advantage of the controllability of light intensity, tumor growth would be inhibited to a certain extent. But oncotherapy triggered by PTT requires combination with a photosensitizer to help achieve photothermal conversion,^13^ and the poor targeting ability of the photosensitizer and limited deep tissue penetration of light restrict its efficacy and further application.^14,15^ Additionally, it is usually difficult to achieve satisfactory results with PTT alone. Therefore, combining PTT therapy with other therapies is an increasingly popular option, aiming to kill tumor cells in deep tissues without damaging surrounding tissues as well as inhibiting tumor recurrence and metastasis.^16^

Recently, bacteria-mediated tumor therapy has attracted more and more attention.^17,18^ Self-propelling through some cytokines produced by tumors, facultative anaerobes could migrate to the immunologically exempt compartment of tumor tissues to escape systemic immune clearance.^19,20^ Taking advantage of hypoxia targeting by bacteria, numerous studies on utilizing bacteria as drug vectors or antitumor vaccines have been reported. After being injected into the body, bacteria specifically colonize tumor tissues and activate the body's innate immune response, accompanied by hindering tumor proliferation without obvious side effects on normal organs.^21–24^ Meanwhile, inflammatory factors will accelerate during bacterial treatment, which could destroy the vessels around the tumor. All these lead to the formation of a thrombus by hemocyte cell extravasation, which darkens the surface of the tumor and increases near-infrared absorption.^25^ These provide support for the photothermal ablation of tumors without photothermal agents. It is reported that a temperature-sensitive plasmid pBV220 could be thermally induced, leading to its non-invasive expression of targeting protein by *in vitro* regulation.^26^ Recently, the application of immune checkpoint inhibitors (ICIs), such as programmed cell death protein 1 (PD1) and cytotoxic T lymphocyte-associated protein-4, has been reported for the treatment of various types of tumors,^27^ which has shown more prominent efficacy in the clinical setting. The type of ICIs used could relieve immunosuppression and boost the immune response of anti-tumor cells.^28–30^ The application of clinical ICIs has some disadvantages, such as low drug utilization rate, obvious side effects, and high cost.^31–33^ Therefore, a great demand remains for seeking alternative solutions to utilize such immunotherapy agents effectively.

In this study, nonpathogenic *Escherichia coli* (*E. coli*) K-12 (MG1655) was selected as a carrier, and temperature-sensitive plasmid pBV220/ClyA-mPD1E-3HA was transferred into the bacteria. We constructed thermally-induced bacteria (TIB), which could express PD1 by heat induction. Taking advantage of the effects of bacteria enriching tumor sites and causing thrombosis, the darkened tumor sites greatly improve the ability to absorb near-infrared light. By controlling light intensity and time, the expression of PD1 was precisely regulated by heat. In tumor tissues, the expressed PD1 on the surface of the bacterial membrane could bind to programmed cell death-ligand 1 (PD-L1) on the tumor cell surface, which could protect T cells from the PD1/PD-L1 immune inhibitory axis. Therefore, PTT combined with TIB-initiated immunotherapy can be strictly manipulated by adjusting the light conditions to inhibit PD-L1-mediated immunosuppression and relieve the inhibition of T cell proliferation. The activated immune system could effectively inhibit tumor cell proliferation, as well as metastasis and diffusion.

## Materials and methods

### Materials

*E. coli* gold and *E. coli* MG1655 (Table S1†) were purchased from Beijing TransGen Biotech Co., Ltd (China). Six-week-old female Balb/c mice were obtained from Three Gorges University (Hubei, China). Mouse colon carcinoma CT26 cells were provided by the China Center for Type Culture Collection (Wuhan, China). Roswell Park Memorial Institute-1640 medium (RPMI-1640), fetal bovine serum (FBS), bovine serum albumin (BSA), trypsin, and phosphate-buffered saline were bought from Invitrogen Corp. Moreover, all other reagents and solvents were used directly after testing their analytical purity.

### Construction of the plasmid

The plasmid pBV220 included a repressor gene TcI, pR–pL operator–promoter gene, and an ampicillin-resistant gene. The pBV220 plasmid was linearized by digestion with restriction enzymes EcoR1 and Sal1. Next, the ClyA-mPD1E-3HA gene was amplified from plasmid pET28a/ClyA-mPD1E-3HA (Chinese Academy of Science, Beijing, China), using synthetic primers which had a 15 nt homology-sequence matching the linearized plasmid (Table S2†). The *E. coli* gold competent cells were transferred the above two fragments for recombination by a T5 recombinant clone. After mass replication, the plasmids were extracted and purified with a plasmid extraction kit (Thermo), then transferred into *E. coli* MG1655. The engineered bacteria were grown on ampicillin-resistant (100 μg mL^−1^) selection Luria–Bertani (LB) agar plates at 37 °C overnight.

### Western blot analysis

Western blot was used to analyze the expression of PD1 protein. Before lysis all samples had 10% SDS and 1% phenylmethylsulfonyl fluoride (PMSF) added. The supernatant was mixed with loading buffer (250 mM Tris–HCl, 10% SDS, 50% glycerin, 0.5% BPB, 5% β-mercaptoethanol, pH 6.8), followed by reaction at 100 °C for 10 min. The protein samples were electrotransferred to a polyvinylidene fluoride membrane after being separated by 10% SDS-PAGE electrophoresis. In addition, blots were incubated in 5% BSA blocking solution, then analyzed using antibodies raised against horseradish peroxidase (HRP, mouse monoclonal antibody, Proteintech, China), HA (rabbit polyclonal antibody, Proteintech, China), or PD-L1 (mouse monoclonal antibody; Proteintech, China), which were diluted 3000 times with 2.5% BSA. Immunoreactive proteins were visualized with western chemiluminescent substrate (Immobilon, USA) as per standard protocols.

### Thermal-induced PD1 expression

The recombinant plasmid pBV220/ClyA-mPD1E-3HA was transferred into *E. coli* MG1655, then analyzed by next-generation sequencing. The positive strain was grown in 1 mL of Luria–Bertani (LB) ampicillin-resistant liquid medium (10 mg mL^−1^ NaCl, 5 mg mL^−1^ yeast extract, 10 mg mL^−1^ tryptone, 10 mg mL^−1^ agarose) overnight at 37 °C, and then diluted to 100 mL with LB medium containing ampicillin until the OD_600_ reached 0.6. Three incubator-shakers with 10 mL cultures were individually kept at 37 °C, 42 °C, and 45 °C for 1 h. For time-dependent experiments, 10 mL cultures were transferred to an incubator-shaker at 37 °C when the OD_600_ reached 0.6. These cultures were transferred to 42 °C conditions for 0, 10, 20, 40, and 60 min. The expression of PD1 in the bacterial lysis was analyzed by Western blot.

### *In vivo* biodistribution of bacteria

Tumor-bearing mice were intravenously injected at a dose of 10^8^ CFU TIB and fed for 0, 12, 24, 48, and 72 h respectively. After feeding at desired time points, the mice were sacrificed to collect the main organs such as heart, liver, spleen, lung, kidney, and tumor tissues. These samples were weighed, homogenized, and diluted to 1 mg mL^−1^ suspension in sterile PBS, but tumor tissue was serially diluted to 0.1 mg mL^−1^. Bacterial colonies were counted after the tissue suspension (100 μL) was plated on ampicillin-resistant LB solid plates at 37 °C for 24 h.

### Enzyme-linked immunosorbent assays

Mouse blood was harvested at 0, 12, 24, and 36 h after TIB injection, and serum was centrifuged at 2500 g, 4 °C for 30 min. Different vasodilator inflammatory factors, such as BK, His, LC4, and PAF in sera, were analyzed using the indicated ELISA kit (Mlbio, Shanghai, China) following the protocols recommended by the manufacturers.

### Analysis of tumor thrombosis

CT26 tumor-bearing mice were individually intravenously injected with PBS (100 μL), MG1655 (100 μL, 10^8^ CFU), and TIB (100 μL, 10^8^ CFU). A camera was used to record the color changes of the tumor surface 0, 12, 18, and 24 h after injection. To analyze the hemoglobin concentration in the tumor, mice were sacrificed 0, 12, and 24 h after injection, and the tumor tissues were collected, homogenized, and continuously diluted to 10 mg mL^−1^ with ice-cold sterile PBS. An ultraviolet spectrophotometer was used to measure the absorbance at 540 nm. Tumor blood platelet endothelial cell adhesion molecule-1 was detected by immunohistochemical staining of CD31 antibodies.

### Fluorescence imaging

Bacteria TIB and MG1655 were cultured until OD_600_ reached 0.6. 1 mL of bacteria medium were centrifuged at 5000 rpm for 2 min, then washed with PBS. The collected cells were resuspended in 1 mL of PBS with 200 μL of DiR solution at 37 °C for 20 min away from light. The labeled bacteria were intravenously injected into the CT26 tumor-bearing mice (200 μL per mouse). The fluorescence signals at the tumor sites at 0, 4, 12, 24, and 36 h were recorded with a fluorescence imager. For a parallel group, the mice were sacrificed, and their main organs and tumor tissues were collected for individual fluorescence analysis.

### Tumour animal model

Six-week-old female Balb/c mice were obtained from the Laboratory Animal Center, of China Three Gorges University. All animal procedures were performed in accordance with the Guidelines for Care and Use of Laboratory Animals of Wuhan University and approved by Institutional Animal Care and Use Committee (IACUC) of the Animal Experiment Center of Wuhan University (Wuhan, China). After seven days of adaptive feeding, the 7 week-old female Balb/c mice gained their normal weight again. The mice were injected at a dose of 5 × 10^5^ CT26 tumor cells on the right buttock. Animals were used in the experiments until the tumor volume reached approximately 100 mm^3^ (tumor volume = 1/2 × length × width^2^).

### Photothermal imaging *in vivo*

The CT26 tumor-bearing mice were fed until the tumor volume reached 100 mm^3^, then intravenously injected with MG1655 (100 μL, 10^8^ CFU), and TIB (100 μL, 10^8^ CFU) individually. 100 μL of PBS was injected for the negative control experiment. 24 h later, a near-infrared laser (808 nm, 1.0 W cm^−2^) was utilized to irradiate the mouse tumor site for 6 min, and the changing temperatures at the tumor sites were recorded with an IR thermal camera.

### *In vivo* anti-tumor efficacy

Female Balb/c tumor-bearing mice were randomly divided into five groups (5 mice per group) and given different treatment regimens: (1) PBS (100 μL PBS); (2) MG1655 (100 μL MG1655, 10^8^ CFU per mouse); (3) MG1655 + L (100 μL MG1655, 10^8^ CFU per mouse); (4) TIB@PD1 (100 μL TIB@PD1, 10^8^ CFU per mouse); and (5) TIB + L (100 μL TIB, 10^8^ CFU per mouse). For the laser irradiation groups, the tumor regions were irradiated with an 808 nm laser (1.0 W cm^−2^, 8 min) 24 h after injection. Body weight and tumor volume in each group were recorded every day with electronic scales and vernier calipers. At the end of the treatment, the mice were sacrificed, and the main organs such as heart, liver, spleen, lung, kidney, and tumor tissue were collected for H&E and immunohistochemical staining.

### Flow cytometry analysis

After 3 days of treatment, the peripheral blood of the mice was extracted. 200 μL of peripheral blood was transferred to a 1.5 mL microcentrifuge tube and incubated with 600 μL of erythrocyte lysate solution for 15 min at 4 °C. Cells were harvested at 500 g, 4 °C for 10 min, then resuspended in 400 μL of erythrocyte lysate buffer. 15 min later, the collected cells were resuspended and washed with 500 μL of PBS (500 g for 5 min), then enumerated by cell-count boards and diluted to 5 × 10^5^ cells mL^−1^. The cells were incubated for 1 h at room temperature with 300 μL of sealing buffer (PBS solution with 0.5% BSA + 10% FBS). Fluorescent antibodies such as CD3, CD8a, CD11b, and CD49b (Anti-mouse Rat monoclonal, Proteintech, China) were added following the instructions, and no antibody was used as a negative control. All samples were incubated at room temperature for 1 h, then collected at 500 g for 5 min. After being washed twice in 1 mL of incubation buffer (PBS solution with 0.5% BSA), the cells were centrifuged at 500 g for 5 min, then the pellet was resuspended in 300 μL of PBS buffer. All data analysis was performed using Flowing Software, and gating for displayed images utilized FlowJo_V10 (FlowJo, LLC).

### Pulmonary metastasis model

The CT26 tumor-bearing mice were treated by different strategies. 3 days later, 5 × 10^5^ CT26 tumor cells were intravenously injected into the mice. After 11 days of normal diet, the mice were sacrificed. Then the lung tissue was stripped for H&E staining to evaluate the pulmonary metastasis.

### Bio-safety assays *in vivo*

Female Balb/c mice were intravenously injected at a dose of 10^8^ CFU TIB. Blood samples were collected for blood parameter assays 0, 12, 24, 72, and 168 h after intravenous injection. To evaluate the function of the liver and kidney after intravenous injection, the peripheral blood of the mice was collected at 0, 12, 24, 72, and 168 h; then biomarkers such as AST, ALT, GGT, CRE, GLU, and urea were tested with an automatic biochemistry analyzer.

### Statistical analysis

The data were presented as the mean ± standard deviation (SD), and the statistical significance was calculated *via* one-way analysis of variance (ANOVA) and Tukey post hoc tests. All statistical analyses were performed using GraphPad Prism 8.0.1 statistical analysis software. A *p*-value <0.05 was considered statistically significant. *p < 0.05; **p < 0.01; ***p < 0.001.

## Results and discussion

### The construction and induced expression of TIB

**Fig. 1:**
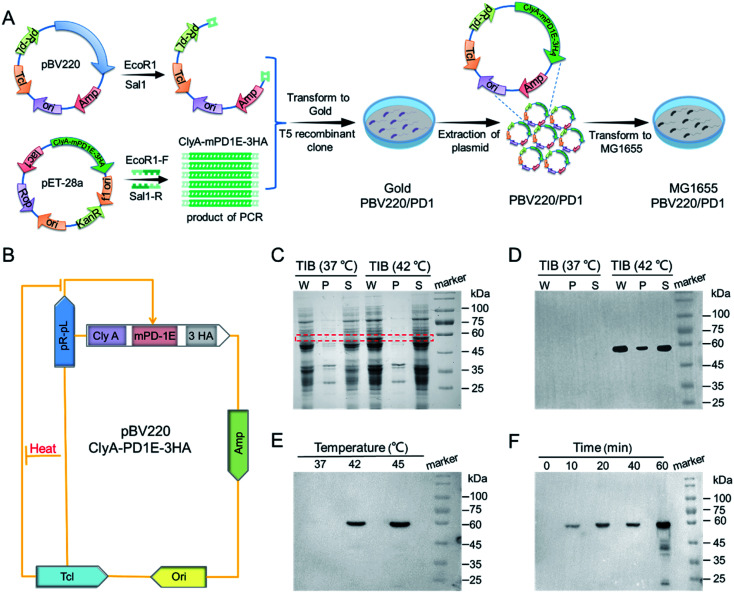
The construction and induced expression of TIB. (A) The construction of plasmid pBV220/ClyA-mPD1E-3HA and engineered bacteria TIB. (B) The mechanism of PD1 expression based on plasmid pBV220. (C) SDS-PAGE analysis of the expression of PD1 by TIB at 37 °C and 42 °C; red box: target protein bands; W: whole cell lysate; P: precipitation of cell lysate; S: supernatant of cell lysate. (D) Western blot used to identify the expression of PD1 by TIB at 37 °C and 42 °C. (E) Temperature-dependent PD1 protein expression in MG1655. (F) Time-dependent expression of PD1 protein at 42 °C.

The construction of TIB is shown schematically in Fig. 1A. A custom-designed plasmid pBV220, containing temperature-sensitive bacteriophage λ repressor cI857 (TcI) and tandem pR–pL promoter, was first constructed. When the temperature is under the threshold value, the repressor protein TcI will be released to prevent pR–pL promoter and downstream protein from expressing. Recombinant protein gene ClyA-mPD1E-3HA genetically fused the bacterial outer membrane protein (ClyA) and mouse PD1 ectodomain protein (mPD1E) and contained three C-terminal hemagglutinin (HA) tags. Therefore, mPD1E protein could be displayed on the surface of the bacteria, which served as an ICI to activate T cells for tumor cell killing. ClyA-mPD1E-3HA was mass replicated by a polymerase chain reaction (PCR) program using synthetic primers, then linked with plasmid pBV220 by a T5 recombinant clone and transformed into *E. coli* gold for the mass replication of plasmids pBV220/ClyA-mPD1E-3HA. Due to the serious inflammatory response caused by common Gram-negative bacterial endotoxins, *in vivo* biosecurity is the primary consideration for a bacteria-based tumor treatment platform. Gene knockout bacteria MG1655, producing fewer lipopolysaccharides than the wild-type, is extensively used for clinical application research due to its noninvasive commensal feature. Therefore, the plasmids were finally transformed into *E. coli* MG1655 after extraction, accompanied by activation of the expression of PD1 by heat.

A schematic diagram of the thermally-induced expression of PD1 *via* plasmid pBV220 is exhibited in Fig. 1B. To confirm that the recombinant protein PD1 (60 kDa) could be expressed by MG1655 when the temperature exceeds the threshold, a sodium dodecyl sulfate-polyacrylamide gel electrophoresis (SDS-PAGE) assay was used for analysis (Fig. 1C). Under the incubation condition of 37 °C, no target protein band was resolved by SDS-PAGE. However, a significant target protein band appeared when incubated at 42 °C, which agreed well with the molecular weight of the target protein ClyA-mPD1E-3HA. Western blot was used to further confirm our judgment, and the strongest expression of PD1 appeared at 42 °C (Fig. 1D). To verify the thermally-induced TIB, the expression of PD1 at different temperatures was detected by Western bolt assay. All those samples were induced by different ambient temperatures for 1 h and, after fragmentation and extraction steps, the solution of PD1 proteins was obtained. As shown in Fig. 1E, the expression of PD1 changed conspicuously when the culture temperature increased. There was no expression at 37 °C and it began to express at an ambient temperature of 42 °C. In particular, when the incubation temperature reached 45 °C, the expression of PD1 would be more than twice as much as that at 42 °C. The contrast experiments confirmed the feasibility and reliability of the plasmid we designed. Next, we explored the expression level of PD1 cultured at 42 °C by sampling at different time points (Fig. 1F). After being incubated for 10 min or more, the PD1 protein expressed by TIB could be detected. Remarkably, the thermally-induced bacteria we designed could accurately regulate the expression of PD1 by ambient temperature and incubation time.

### The mechanism of TIB *in vivo*

**Fig. 2:**
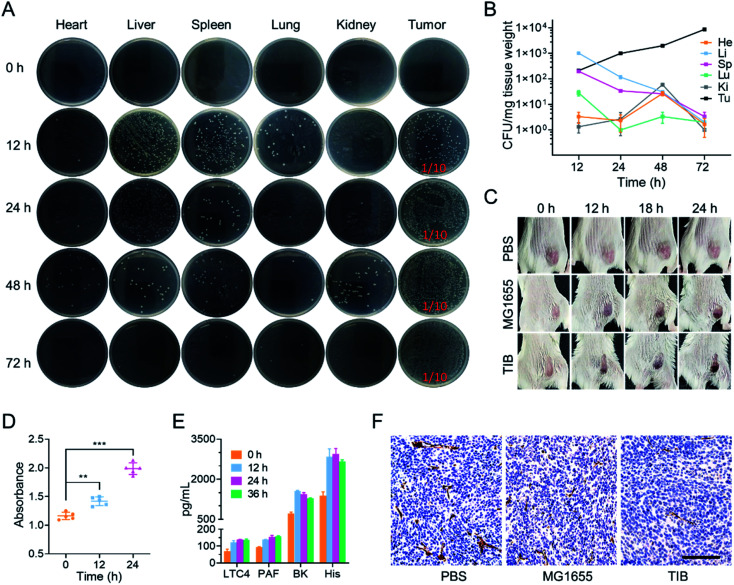
Mechanism of TIB *in vivo*. (A) Representative photographs of LB agar plates showing the CFU of TIB in various organs and tumor tissues of mice. (B) A plot showing TIB accumulation in various organs and tumor tissues of mice; He: heart; Li: liver; Sp: spleen; Lu: lung; Ki: kidney; Tu: tumor. (C) The color changes of the tumor surface recorded using a camera. (D) Hemoglobin enrichment at tumor sites at different times after TIB injection. (E) Concentrations of LTC4, PAF, BK, and His in mice serum measured *via* ELISA 0, 12, 24, and 36 h after TIB inoculation. (F) The microvascular density of tumors observed based on immunohistochemical sections; scale bar = 100 μm.

Next, we explored the behavior of TIB in CT26 tumor-bearing mice by intravenous injection at a dose of 10^8^ colony-forming units (CFU) per mouse. 0, 12, 24, 48, and 72 h after injection, the mice were sacrificed to harvest the main organs and tumor tissues. After homogenization, dilution, and plating onto an LB agar plate, the number of bacterial colonies on each plate was counted (Fig. 2A). Interestingly, we found that the number of TIB in the major organs was gradually eliminated. In marked contrast, the bacteria in tumor tissues steadily increased over time (Fig. 2B). We speculate that the tumor tendency of facultative anaerobic bacteria and the eutrophication tumor microenvironment caused this phenomenon.

It is reported that tumor sites would turn to an obviously darkened color a few hours after injecting bacteria into tumor-bearing mice.^24^ In our experiment, CT26 tumor-bearing mice were intravenously injected with TIB and MG1655 at an equal dose of 10^8^ CFU per mouse, and mice injected with 100 μL phosphate-buffered saline (PBS) were used as a negative control trial. At predetermined time points after injection, the color changes of the tumor sites were recorded with a camera (Fig. 2C). It was interesting that the color of the tumor surface continuously deepened after bacteria were inoculated, whereas there were no changes in the PBS group. Therefore, colonization of bacteria within the tumor may mediate tumor-specific thrombosis. To confirm our conjecture that the darkening of the tumor was caused by thrombogenesis, the tumor tissues were collected after intravenous injection with TIB at different time points to test the level of hemoglobin. As recorded at an absorption value of 540 nm by an ultraviolet spectrophotometer, the hemoglobin concentrations in a tumor suspension of bacteria-injected mice were significantly increased within the monitoring time (Fig. 2D). Thrombosis associated vasodilator inflammatory factors such as leukotriene C4 (LC4), bradykinin (BK), platelet-activating factor (PAF), and histamine (His), which could strengthen the capillary permeability and dilate the vascular aperture, were further detected with an enzyme-linked immunosorbent assay (ELISA). As shown in Fig. 2E, the concentrations of all cytokines increased significantly after bacterial injection. These elevated cytokines enticed blood to spread out of the extravascular area, resulting in thrombosis with the help of platelet activation. Moreover, we observed tumor vascular destruction by immunohistochemical staining with CD31 antibody. CD31, a marker of blood vascular endothelial cells, could be used to evaluate vascular integrity. Fig. 2F depicts the fact that the area of vessels labeled by CD31 antibody in the PBS group was significantly higher than that in other bacterial injection groups, and further statistics showed that there was no significant difference in vascular area between group MG1655 and group TIB (Fig. S1†), indicating that tumor vessels were damaged after injection of bacteria. Therefore, intratumoral bacterial infection and colonization would trigger intratumoral thrombosis by disrupting tumor vessels, which in turn would darken the tumor surface. The blackened area could enhance the absorption of NIR and cause an extreme improvement in the photothermal conversion efficiency to provide support for subsequent photothermal therapy. After intratumoral thrombosis, the number of bacteria could still increase, so the formation of an intratumoral thrombus has a positive effect on the photothermal treatment of bacteria.

### TIB-mediated photothermal therapy

**Fig. 3:**
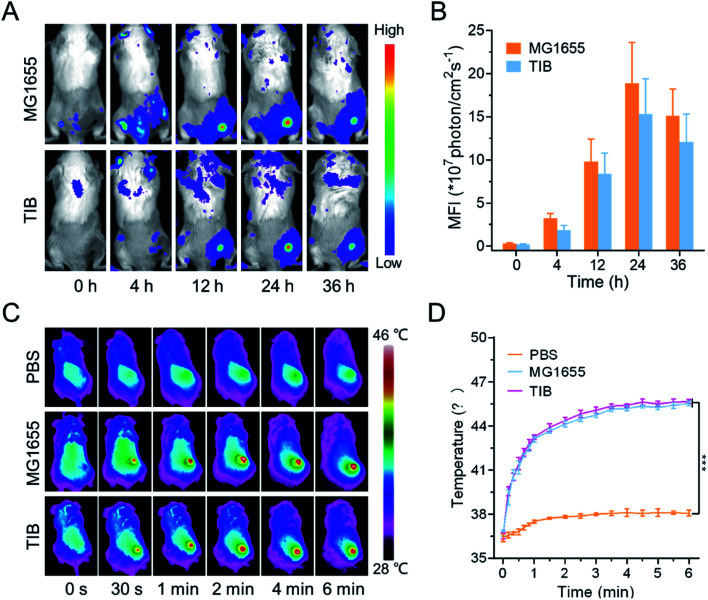
Photothermal therapy mediated by TIB. (A) Time-dependent *in vivo* fluorescence imaging of CT26 tumor-bearing mice inoculated with DiR-labeled TIB and (B) the corresponding MFI of each group. (C) Time-dependent photothermal imaging of MG1655 TIB at tumor sites. (D) The change in temperature over time at tumor sites in different groups under 808 nm NIR irradiation (1 W cm^−2^).

The biodistribution of bacteria *in vivo* could be visualized by fluorescence imaging. In our study, the cell membrane was labeled with 1,1-dioctadecyl-3,3,3,3-tetramethylindotricarbocyanine iodide (DiR) to evaluate the tumor tendency of TIB. As shown in Fig. 3A, after intravenous injection of DiR-labeled MG1655 and TIB, the strong DiR fluorescence intensity was visualized in the tumor site over time, revealing the tumor-targeting ability of the bacteria. 24 h later, a strong fluorescence signal could be detected in the tumor site (Fig. 3B). At the same time, the parallel group of mice was sacrificed to collect the main organs containing heart, liver, spleen, lung, kidney, and tumor tissues for mean fluorescent intensity (MFI) analysis (Fig. S2†). We observed a stronger fluorescence signal at the tumor tissues both in DiR-labeled MG1655 and TIB groups, indicating that bacteria could be especially enriched at the tumor sites. Based on the marked tumor-targeting ability of bacteria and the darkened color on the surface of tumor showing satisfactory NIR absorbance, we next evaluated the conversion ability of intratumoral photothermal therapy with an IR camera. The tumor-bearing mice were individually injected with MG1655 and TIB, and PBS was used as the negative control. 24 h later, 808 nm laser irradiation (1 W cm^−2^) was used to irradiate the site of the mice tumor for 6 min (Fig. 3C). Compared with the PBS group, the temperature of the tumor sites in both MG1655 and TIB groups was increased to about 46 °C under laser irradiation, showing gratifying photothermal conversion efficiency (Fig. 3D). These results supported the feasibility of bacteria-mediated tumor photothermal therapy *in vivo* based on the formation of a thrombus, which could activate the expression of PD1 when the temperature is over 42 °C under laser irradiation.

### Immune system activation by TIB@PD1 *in vivo*

In addition, the expression of PD1 would display on the surface of TIB (TIB@PD1) after being thermally-induced. We tested the effectiveness of TIB@PD1 combined with CT26 tumor cells. The results showed that tumor cells could combine with TIB@PD1 but not MG1655, and this phenomenon might be caused by the binding of PD1 to PD-L1 on the tumor surface (Fig. S3†).

**Fig. 4:**
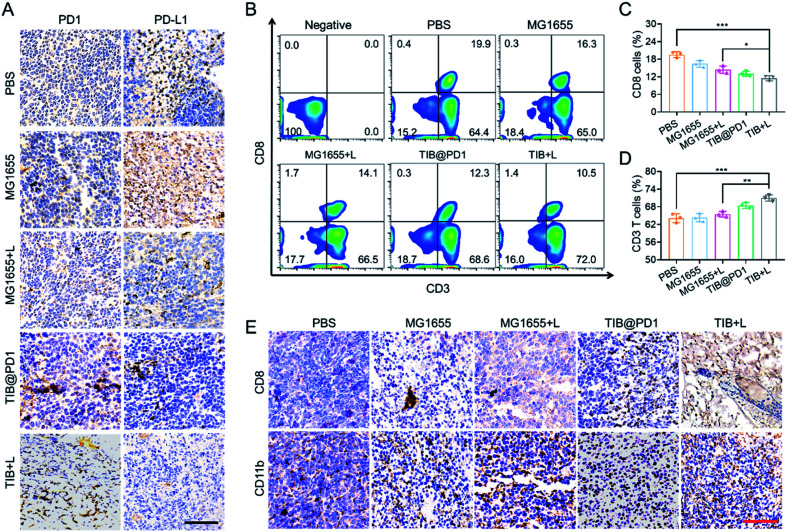
Immune system activation by TIB@PD1 *in vivo*. (A) Immunohistochemical images showing the proteins of PD-L1 and PD1 in CT26 tumor tissue (brown); scale bar = 100 μm. (B) Flow cytometry showing the T cell distribution in CT26 tumor-bearing mouse blood. (C) Corresponding quantitative analysis of CD3^+^ T cells in CT26 tumor-bearing mouse blood. (D) Corresponding quantitative analysis of CD8^+^ T cells in CT26 tumor-bearing mouse blood. (E) Immunohistochemical images showing CD8^+^ T cells and CD11b^+^ cells in different groups of CT26 tumor-bearing mouse tumor tissue (brown); scale bar = 100 μm.

The immune checkpoint receptor protein PD1 could concatenate PD-L1 on the surface of tumor cells and protect the cells from immune attack. To verify that TIB@PD1 could affect the PD1/PD-L1 immune inhibitory axis in tumors, tumor tissues of mice in each group were stripped after treatment, and immunohistochemical staining of PD1 and PD-L1 were verified (Fig. 4A and S4†). The level of PD-L1 within the CT26 tumor tissues was significantly higher in the PBS and MG1655 groups, but almost not observed in the TIB + L group. We speculated that this might be related to the shielding of the PD-L1 target by PD1. According to the immunohistochemical staining of PD1, it could be seen intuitively that the level of PD1 was significantly increased within the tumor tissues in the TIB@PD1 and TIB + L groups. These phenomena might be caused by the expression of PD1 after NIR irradiation and then block the PD1/PD-L1 axis. CD8^+^ T cells would be highly limited by inhibitory receptor proteins, such as PD-L1, which could induce the dysfunction of T cells by interacting with receptor protein PD1 on the surface of T cells. To confirm that immune evasion could be inhibited by our strategy, flow cytometry was used to analyze the peripheral blood cells of tumor-bearing mice after three days of treatment (Fig. 4B). Compared with the PBS group, we observed that the percentage of CD8^+^ T cells in the TIB@PD1 and TIB + L groups decreased significantly. Given tumor immune evasion, the body immune system might produce more CD8^+^ T cells to attack tumor cells in the PBS group. With the improvement in the efficiency of CD8^+^ T cells strangling tumor cells, the percentage of CD8^+^ T cells in the TIB@PD1 and TIB + L groups recovered to a low level (Fig. 4C). On the other hand, the percentage of CD3^+^ T cells in TIB + L-treated mice was much higher than that in the PBS and MG1655 groups (Fig. 4D). Next, we studied whether the innate immune system could be activated after the treatment. The proportions of CD11b^+^ cells (macrophages) and CD49b^+^ cells (NK cells) were analyzed by flow cytometry (Fig. S5†). Compared to the PBS group, the percentage content of CD11b^+^ cells and CD49b^+^ cells obviously increased in the group injected with MG1655 then subjected to near-infrared light irradiation for 8 min (MG1655 + L), TIB@PD1, and the group injected with TIB then subjected to near-infrared light irradiation for 8 min (TIB + L). Considering that receptor protein PD1 was highly exhausted on the surface of CD8^+^ T cells at the tumor site, the PD1/PD-L1 axis was blocked with the expression of PD1, which could enhance the infiltration of CD8^+^ T cells. Fig. 4E demonstrates this phenomenon by immunohistochemical staining of CD8 of tumor sections. Immunohistochemical staining of CD11b supported the idea that our treatment could also enhance macrophage cell infiltration (Fig. S6†). These results indicated that the adaptive immune response and innate immunity were activated by TIB-mediated photothermal therapy, and the expression of PD1 would block the PD1/PD-L1 pathway-mediated immunosuppression.

### Bacterial therapy *in vivo*

**Fig. 5:**
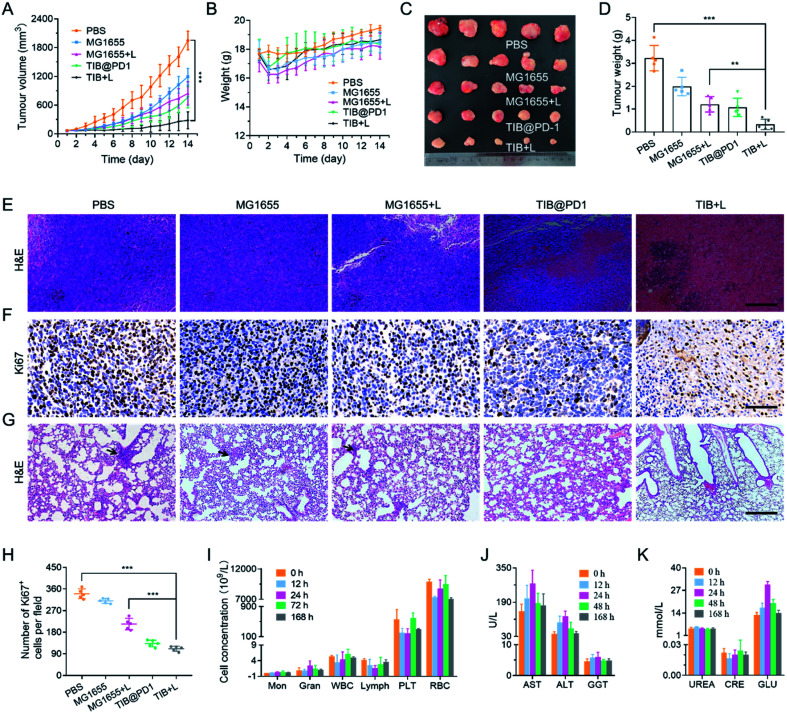
Bacterial therapy *in vivo*. (A) Tumor volume growth over time for mice in different groups. (B) Mouse body weights in different groups over time. (C) A photograph of excised tumor tissue after different treatments. (D) The final tumor weights after receiving different treatments. (E) Tumor H&E staining in different groups; scale bar = 100 μm. (F) Immunohistochemical staining of Ki67 in different groups (brown); scale bar = 100 μm. (G) H&E staining of lung sections; black arrows indicate tumor metastasis; scale bar = 50 μm. (H) The number of Ki67^+^ cells in different treatment groups at tumor sites. (I) Routine analysis of blood from treated mice 12, 24, 72, and 168 h after caudal vein injection. (J and K) Blood biochemical analysis was performed using tumor-bearing mice after 12 h, 24 h, 48 h, and 168 h of TIB treatment.

Based on the above analysis, we believe that bacteria-based tumor therapy could achieve a good antitumor effect *in vivo*. According to the therapeutic schedule in Fig. S7,† we next evaluated the *in vivo* antitumor effect through the CT26 tumor-bearing mice model. There were 5 mice in each group, and MG1655, TIB, and TIB@PD1 were injected into the blood vessels of mice through the tail vein at an equal dose of 10^8^ CFU per mouse. For PTT groups, 24 h post-injection, the mice were exposed to an 808 nm laser (1 W cm^−2^) for 8 min to generate heat for PTT and induced expression of PD1 at the same time. The tumor volume and body weight of the mice were recorded over the following 14 days (Fig. 5A and S8†). Compared with the average tumor volume in the PBS group, tumor proliferation was inhibited to different degrees in the groups MG1655, MG1655 + L, and TIB@PD1. Whereas the tumor volume of mice treated with TIB + L was controlled below 300 mm^3^ at the end of treatment. These results demonstrated that the additional expression of PD1 induced by PTT could further weaken the tumor-specific PD-L1 blockade effect *in vivo* and trigger T cells to attack tumor cells. During the 14 days of treatment, the body weight fluctuated slightly at the beginning of treatment but recovered subsequently (Fig. 5B and S9†). At the end of the treatment, the mice were euthanized to harvest the tumor tissues for photography (Fig. 5C). Meanwhile, the tumor tissues were weighed and counted in Fig. 5D, which also confirmed the above results. Finally, the tumor tissues were sliced for hematoxylin–eosin (H&E) staining (Fig. 5E) and Ki67 immunohistochemical staining (Fig. 5F). Widespread nuclear pyknosis and karyolysis appeared in H&E staining after treatment by TIB@PD1 and TIB + L, revealing extensive damage to and death of tumor cells. Meanwhile, the results of Ki67 immunohistochemical staining showed fewer proliferating cells in the TIB + L group (Fig. 5H). Compared to the TIB@PD1 and MG1655 + L groups, these proofs expressly certified the improved therapeutic effect of tumor-bearing mice treated by TIB + L, which was mainly caused by the expression of PD1 in conjunction with PTT. The blockage of PD1/PD-L1 pathway-mediated tumor immune evasion by PD1 could enhance CD8^+^ T cell infiltration and immunological memory. We verified our hypothesis by establishing a lung metastasis model, and the experimental results are shown in Fig. 5G and S10.† After three days of therapy, the tumor-bearing mice were intravenously injected at a dose of 5 × 10^5^ CT26 tumor cells. At the end of treatment, each mouse was sacrificed and stripped of lung tissue for H&E staining to evaluate the anti-metastasis ability. These data showed that pulmonary metastasis was observed in the PBS, MG1655, and MG1655 + L groups, but almost not observed in the TIB@PD1 and TIB + L groups.

Biosafety is a wide concern for bacteria-mediated tumor therapy, so we evaluated many aspects of safety. Firstly, to evaluate the main blood cell concentration, blood samples were collected at 0, 12, 24, 72, and 168 h after intravenous injection for blood parameter assays. As shown in Fig. 5I, all these biomarkers fluctuated around a normal level. Next, the function of the liver and kidney were evaluated by blood biochemistry assays, with mouse serum extracted at 0, 12, 24, 48, and 168 h after intravenous injection. Liver-function-related proteases, like aspartate aminotransferase (AST), alanine transaminase (ALT), and gamma-glutamyl transpeptidase (GGT), in the treatment group were slightly elevated for the first 24 h after injection and then tended to be normal. After 48 h, these relative proteases recovered to a normal range (Fig. 5J). Meanwhile, kidney function was explained by biomarkers like urea and creatinine (CRE). The level of relative biomarkers was in a normal fluctuation range. The relative concentration of glucose (GLU) was slightly raised on the first day and then tended to be normal (Fig. 5K). These blood biochemical tests supported negligible side effects from our treatment when treated *in vivo*. At the end of treatment, the main organs like the heart, liver, spleen, lung, and kidney were stripped for H&E staining, and no pathological changes were observed from H&E (Fig. S11†). The above results supported bacteria-mediated tumor therapy *in vivo* having better biosafety and therapeutic efficacy.

## Discussion

In this study, we broke with the traditional way of overcoming the obstacles of low drug utilization efficiency and side effects caused by systemic administration. Thermally-induced bacteria were constructed, which were combined with a temperature-sensitive plasmid pBV220 to initiate a downstream gene under temperature regulation. In our treatment strategy, bacteria could gather at the tumor site and cause thrombosis, and the expression of the targeting protein PD1 could be activated by photothermal therapy, which in turn triggered immune activation *via* protecting T cells from the PD1/PD-L1 immune inhibitory axis. In particular, this strategy showed obvious biosafety and initiated PTT in the absence of a photosensitizer. Utilizing the body's immune system to interfere with the development of tumors provides a promising method for tumor treatment.

## Conflicts of interest

There are no conflicts to declare.

## Supplementary Material

NA-004-D1NA00857A-s001
