# Supplementary material for: Bacteria-mediated tumor immunotherapy via photothermally-programmed PD1 expression
Source: Nanoscale Adv. 2022 Feb 7;4(6):1577–86. doi: 10.1039/d1na00857a (PMC9417531; doi:10.1039/d1na00857a)
Supplement: NA-004-D1NA00857A-s001 [file NA-004-D1NA00857A-s001.pdf]

Electronic Supplementary Material (ESI) for Nanoscale Advances.
This journal is © The Royal Society of Chemistry 2022

Bacteria-mediated tumor immunotherapy via photothermally-programmed PD1 expression

Wenxuan Xu,[1,‡] Debao Ren,[1,‡] Zimeng Yu,[1] Jia Hou,[1] Fan Huang,[1] Tingfang Gan,[1] Ping Ji,[2] Cheng Zhang,[1] Lixin Ma[1,*] Yunhong Hu[1,*]

[1]State Key Laboratory of Biocatalysis and Enzyme Engineering, Hubei Key Laboratory of Industrial Biotechnology, School of Life Sciences, Hubei University, Wuhan 430062, P.R. China

[2]Department of Chemistry, Wuhan University, Wuhan, 430072, P.R. China

[‡]These authors contributed equally to this work

*Corresponding author E-mail: malixing@hubu.edu.cn; huyunhong@hubu.edu.cn

Table S1. Bacterial strains used in this study

Bacteria strain	Plasmid	Use
Gold	pBV220/ClyA-mPD1E-3HA	Consteuction of recombinant plasmid
MG1655	pBV220/ClyA-mPD1E-3HA	Expression of PD1

Table S2. Primers used to constructed pBV220/ClyA-mPD1E-3HA

Number	Name	Sequence (5' to 3')
1	ePD1-5'EcoRI	TTAAAAATTAAGGAGGAATTCATGACTGAAATCGTTGCAGA
2	ePD1-3'SalI	ACAGAAGCTTGGCTGCAGGTCGACTTAGGCATAGTCGGGGACA

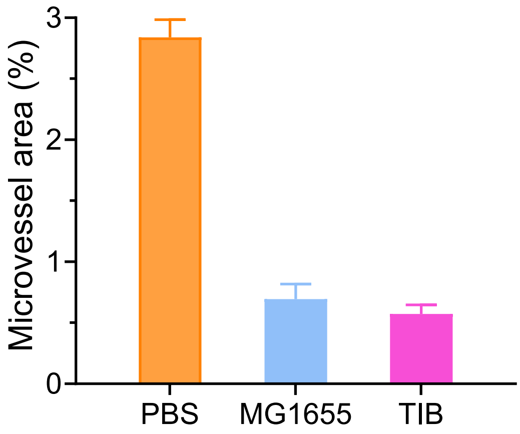

Fig S1 Statistical of tumor microvascular area of mice treated with different strategies.

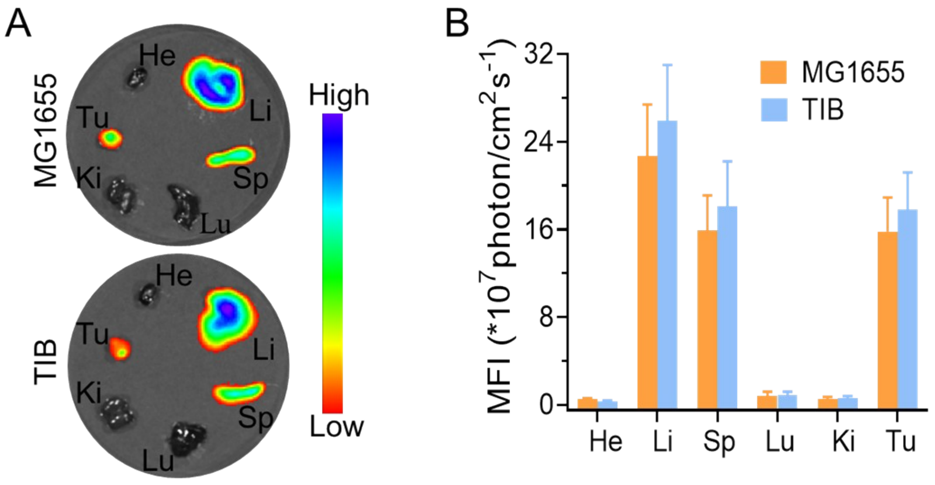

Fig S2 (A) Fluorescence imaging of individual tissues after inoculation of bacteria in tail vein for 24 h (He-heart, Li-liver; Sp-spleen, Lu-lung, Ki-kidney, and Tu-tumor). B) Corresponding MFI of isolated organs with tumor tissue.

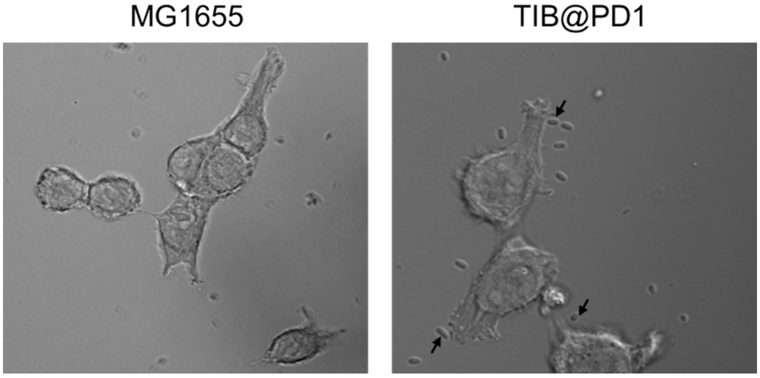

Fig S3 Bacterial TIB@PD1 combine with CT26 tumor cells**.**

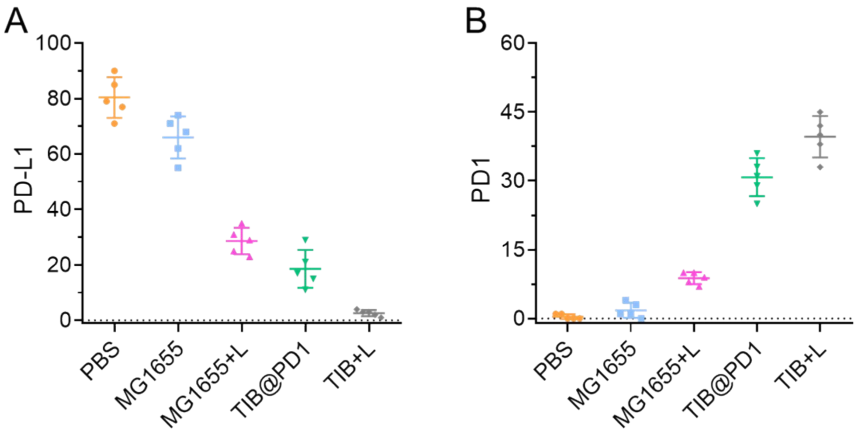

Fig S4 (A) The number of PD-L1 labeled cells at tumor site per field. (B) The number of PD1 labeled cells at tumor site per field.

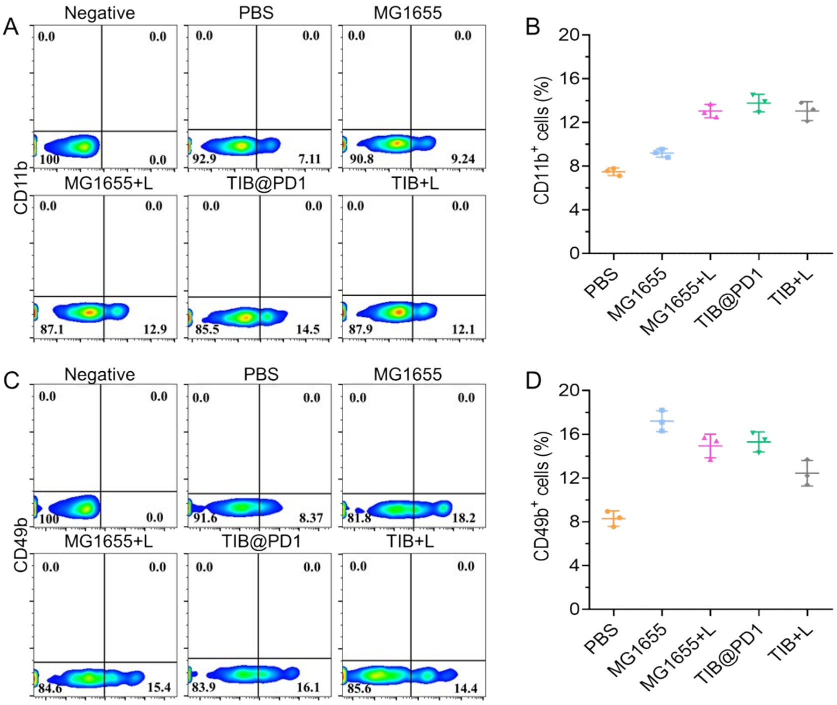

Fig S5 (A) Flow cytometry showing the distribution of CD11b^{+} cells in the CT26 tumor-bearing mouse blood. (B) The corresponding quantitative analysis of the CD11b^{+} cells data. (C) Flow cytometry showing the distribution of CD49b^{+} cells in the CT26 tumor-bearing mouse blood. (D) The corresponding quantitative analysis of the CD49b^{+} cells data.

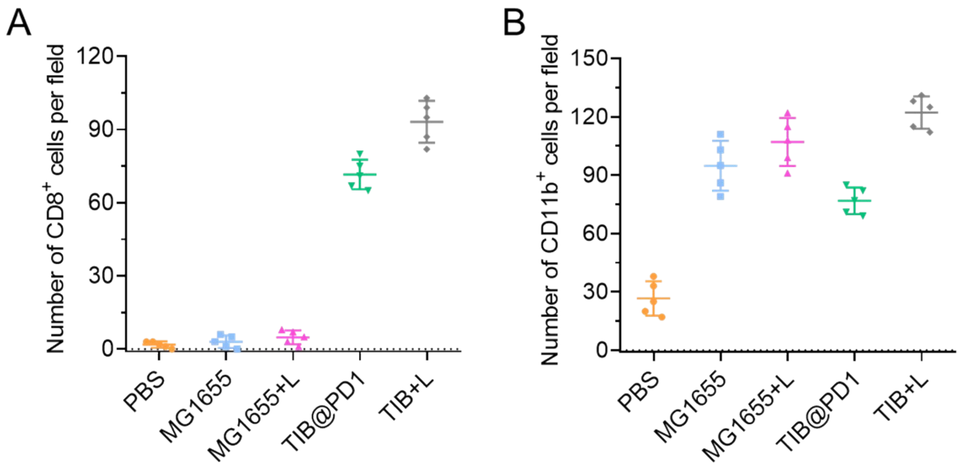

Fig S6 (A) Number of CD8^{+} T cells at tumor site per field. (B) Number of CD11b^{+} cells at tumor site per field.

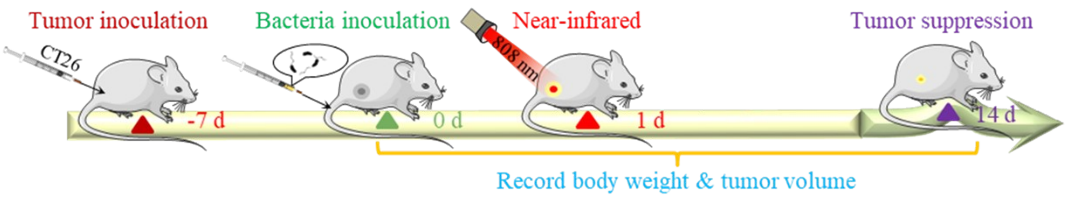

Fig S7 Treatment schedule of CT26 tumor-bearing mice by using bacteria and NIR irradiation.

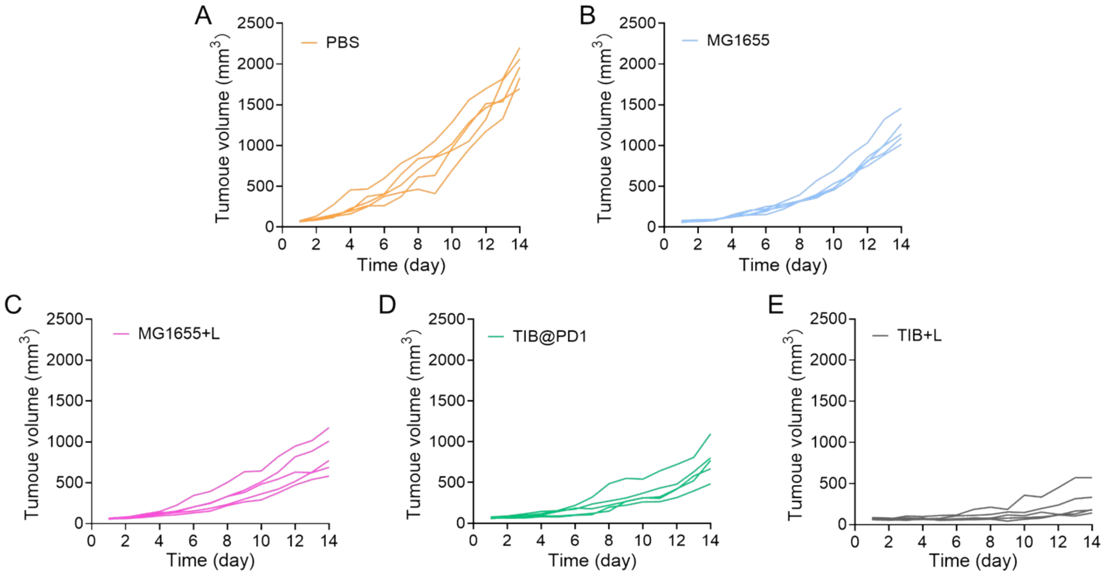

Fig S8 Growth curves of subcutaneously implanted CT26 tumors following treatment with PBS, MG1655, MG1655+L, TIB@PD1, and TIB+L.

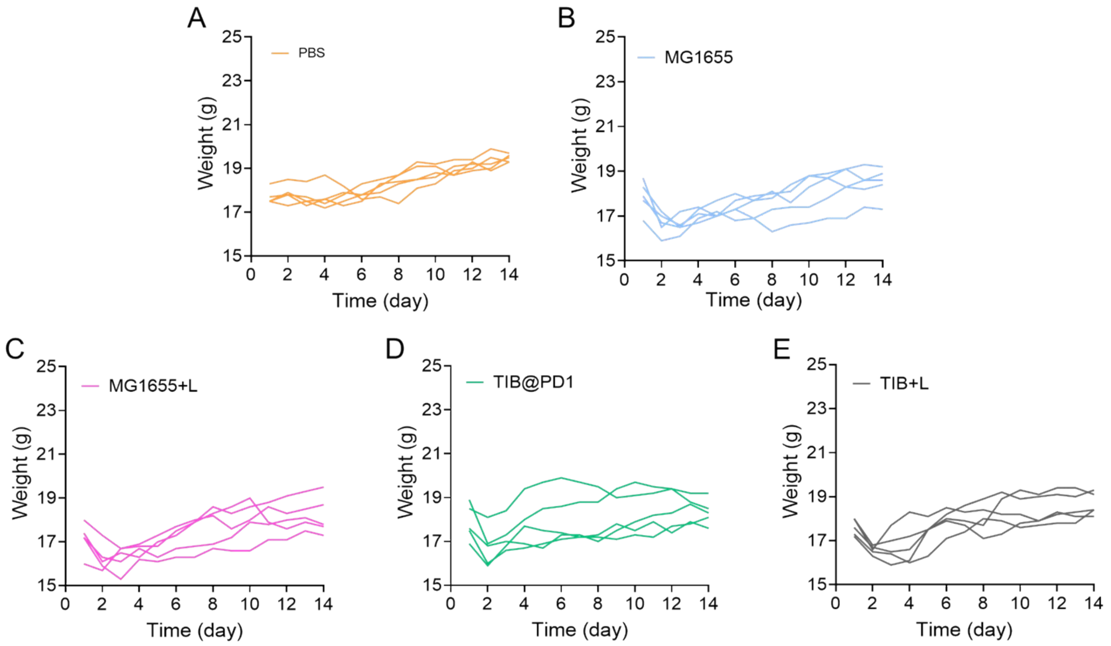

Fig S9 The weight of mice in each group following treatment with PBS, MG1655, MG1655+L, TIB@PD1, and TIB+L.

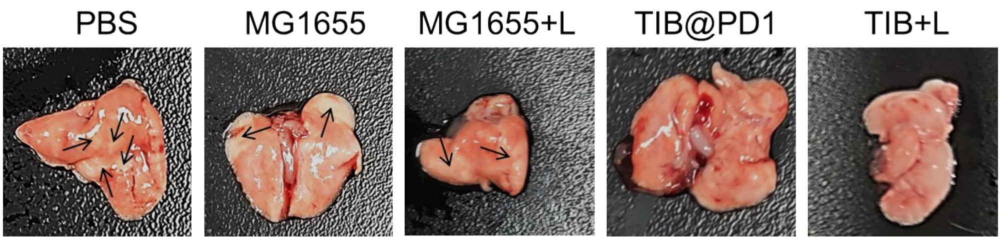

Fig S10 Physical image of the lung of CT26 tumor-bearing mice, arrow shows lung metastasis**.**

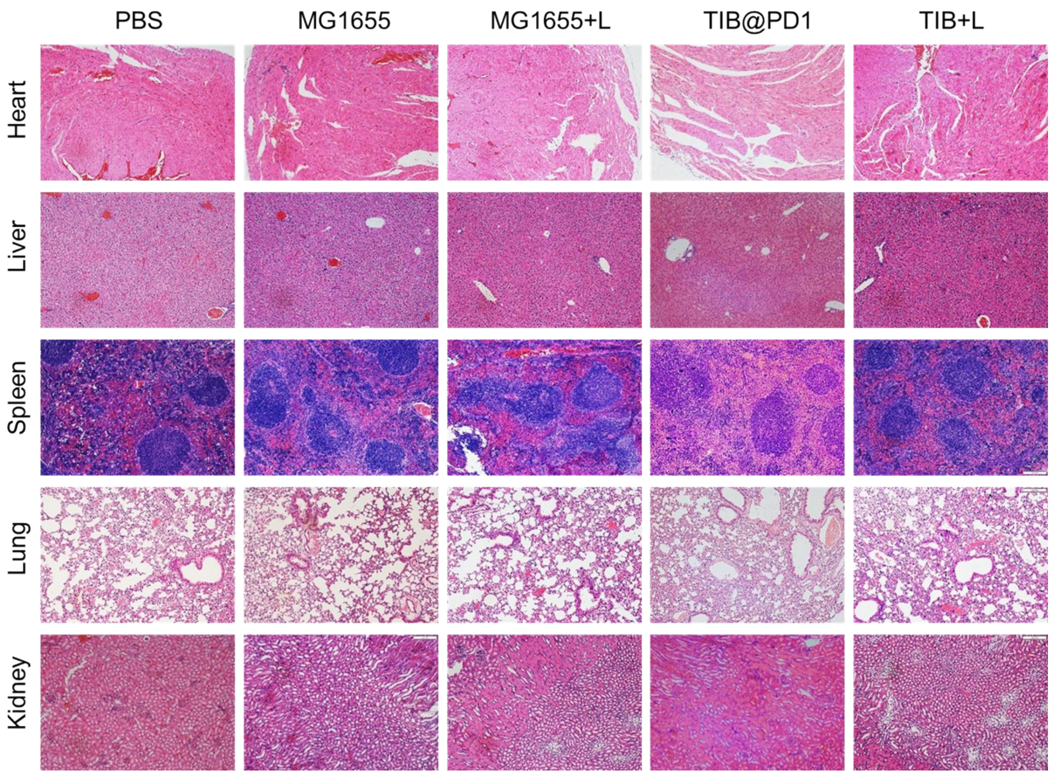

Fig S11 Representative H&E for main organs at the end of the experiments.
